# Improving biocuration of microRNAs in diseases: a case study in idiopathic pulmonary fibrosis

**DOI:** 10.1093/database/bax030

**Published:** 2017-04-22

**Authors:** Yalbi Itzel Balderas-Martínez, Fabio Rinaldi, Gabriela Contreras, Hilda Solano-Lira, Mishael Sánchez-Pérez, Julio Collado-Vides, Moisés Selman, Annie Pardo

**Affiliations:** 1 Facultad de Ciencias, Departamento Biología Celular, Universidad Nacional Autónoma de México, Ciudad Universitaria, Circuito Exterior s/n, Coyoacán, CP 04510, Ciudad de México, CDMX, México; 2 CONACYT-INER Ismael Cosío Villegas, Departamento Investigación, Calzada de Tlalpan 4502 Sección XVI, Tlalpan, CP Ciudad de México, CDMX, México; 3 Swiss Institute of Bioinformatics and Institute of Computational Linguistics, University of Zurich, Andreasstrasse 15, CH-8050 Zurich, Switzerland; 4 Center for Genomics Sciences, Computational Genomics Program, Universidad Nacional Autónoma de México, Av. Universidad s/n, Chamilpa, CP 62210, Cuernavaca, Morelos, México; 5 Instituto Nacional de Enfermedades Respiratorias Ismael Cosío Villegas, Dirección de Investigación Calzada de Tlalpan 4502 Sección XVI, Tlalpan, CP Ciudad de México, CDMX, México

## Abstract

MicroRNAs (miRNAs) are small and non-coding RNA molecules that inhibit gene expression posttranscriptionally. They play important roles in several biological processes, and in recent years there has been an interest in studying how they are related to the pathogenesis of diseases. Although there are already some databases that contain information for miRNAs and their relation with illnesses, their curation represents a significant challenge due to the amount of information that is being generated every day. In particular, respiratory diseases are poorly documented in databases, despite the fact that they are of increasing concern regarding morbidity, mortality and economic impacts. In this work, we present the results that we obtained in the BioCreative Interactive Track (IAT), using a semiautomatic approach for improving biocuration of miRNAs related to diseases. Our procedures will be useful to complement databases that contain this type of information. We adapted the OntoGene text mining pipeline and the ODIN curation system in a full-text corpus of scientific publications concerning one specific respiratory disease: idiopathic pulmonary fibrosis, the most common and aggressive of the idiopathic interstitial cases of pneumonia. We curated 823 miRNA text snippets and found a total of 246 miRNAs related to this disease based on our semiautomatic approach with the system OntoGene/ODIN. The biocuration throughput improved by a factor of 12 compared with traditional manual biocuration. A significant advantage of our semiautomatic pipeline is that it can be applied to obtain the miRNAs of all the respiratory diseases and offers the possibility to be used for other illnesses.

**Database URL:** http://odin.ccg.unam.mx/ODIN/bc2015-miRNA/

## Introduction

MicroRNAs (miRNAs) are small and noncoding RNA molecules that represent a class of regulatory RNAs that decrease mRNA stability or suppress gene expression at a posttranscriptional level (1) playing crucial roles in biological processes. Although they were discovered in 1993 (2), the term miRNA was only introduced in 2001 (3). Therefore, it is not trivial to find articles associated with miRNAs before 2001. After this date, the number of experimental research reports on miRNAs increased rapidly (see online supplementary Figure S1), most of them describing how miRNAs are related to the pathogenesis of human diseases.

At this time, there are about 324 219 known interactions between miRNAs and their target genes for 2619 miRNAs, obtained from a total of 4264 articles about different organs and human diseases in the mirTarBase v6.0 (4), an experimentally validated miRNA-target interactions database. However, some studies have suggested that miRNAs may regulate about 60% of the human encoded mRNAs (5), meaning that the number of known interactions will increase. Adding this information to databases represents a major challenge for biocurators. Although there is an interest in manual biocuration of the miRNAs and miRNA-target interactions associated with diseases (4), extraction of such data still represents a significant challenge due to the amount of information that is being generated every day. Moreover, there are automatic systems to extract miRNA-related reports from the literature (6–15), which all claim to be reliable. Although it is certainly possible to extract mentions of miRNAs from a text in a reliable way, as we also show in the section ‘Regular Expressions’ of this article, it is more difficult to reliably extract related information such as experimental conditions.

For this reason, it has become more important to have a semiautomatic approach to obtain and compile information on interactions associated with the pathogenesis of diseases more efficiently. Especially, respiratory diseases are of increasing concern regarding morbidity, mortality and economic impacts (16). In this article, we describe the results of a pilot project that focused on idiopathic pulmonary fibrosis (IPF).

IPF is the most common and aggressive of the idiopathic interstitial pneumonia, and it is usually progressive, irreversible and lethal (17). The pathogenic mechanisms remain elusive, but the disease is characterized by an aberrant activation of the alveolar epithelial cells, which produce several mediators inducing the migration, proliferation and activation of fibroblasts, which in turn secrete excessive amounts of extracellular matrix components that destroy the normal architecture of the lungs (17). Despite its unknown cause, a growing body of evidence indicates that dysregulation of miRNAs is involved in these processes, and actually, many of them have been shown to be differentially expressed in IPF patients compared with healthy individuals (18).

Despite its importance, in primary databases, it is difficult to find miRNAs associated with respiratory diseases, specifically with IPF. For instance, in the miRTarBase, using the ‘search by disease’ function with the keywords ‘Lung’ or ‘Pulmonary’, there are only a few miRNAs associated with some categories of respiratory diseases ( see online supplementary Table S2). Note that categories are not clearly differentiated by specific illness, e.g. IPF is an interstitial disease, but other interstitial diseases could be included in the category ‘Lung diseases interstitial’. Also, IPF could be in the ‘Pulmonary Fibrosis’ category, but not all of the pulmonary fibrosis diseases are idiopathic.

There are other databases that contain curated information from the literature on miRNAs—miRBase (19), miRecords (20), miRNAmap (21) and TarBase (7)—and some resources that incorporate gene ontology annotations or diseases—miRGator (22), mir2Disease (23) and miRWalk (24); however, in general, annotations related to respiratory diseases are poorly documented, or the information is not up to date.

In this work, we implemented a semiautomatic approach using a text mining system to curate information on miRNAs involved in IPF. This approach was performed in the context of the BioCreative V IAT. BioCreative (Critical Assessment of Information Extraction in Biology) is a community forum which evaluates text mining and information extraction systems that are applied to the biomedical domain (25).

To participate in the BioCreative V IAT, we customized the text mining system OntoGene (26), which is available in a client-server architecture where the remote user can access the results of the annotation pipeline through the browser-based system interface, OntoGene Document Inspector (ODIN) (27). OntoGene, originally developed for the extraction of biomedical entities and their interactions, proved to be quite successful in past BioCreative challenges, e.g. best results in finding protein-protein interactions in BioCreative 2009 (26) and best results in finding gene/disease/chemical relationships in 2012 (28). OntoGene/ODIN has already been used in the curation of different biological elements with reliable results (29).

In addition to the version of ODIN for miRNAs, other versions were provided to biocurators that participated in the IAT: the Comparative Toxicogenomics Database version, customized for extraction of information on genes/diseases/chemicals and their interactions (30), and a RegulonDB version, customized for extraction of genes, transcription factors (TFs), methods, conditions, effects and other concepts (29). In the present work, we discuss only the version for miRNAs, showing the results of the biocuration of 823 miRNA text snippets related to 246 miRNAs associated to IPF. The OntoGene/ODIN system had a very high rate of completion and received the top scores for System Usability Scale (SUS): usability, and learnability in the IAT (31). Our biocuration protocol was 12 times faster than traditional manual biocuration and can be applied to obtain miRNAs of other illnesses.

## Materials and methods

### Obtaining the dataset

We found 66 articles in PubMed associated with the key terms ‘miRNA’ and ‘IPF’. Only 32 full-length articles were free in PubMed Central (PMC). They were selected and converted to text using PDFlib GmbH TET (Text Extraction Toolkit version 4.2; http://www.pdflib.com/) (32) and then cleaned with simple tools, i.e. unwanted characters were removed. This process takes a maximum of five minutes per article. To reduce manual labor, it is also possible to use different formats (XML or HTML) in the cases when they are available or to use the PMC Author article Collection, which has a repository of XML and plain text files for articles published since July 2008, (http://www.ncbi.nlm.nih.gov/pmc/about/mscollection/) (33). Unfortunately, since less than half of the articles that we considered were available in a structured format (from PMC), we could not follow this approach and opted instead for the pdf to text conversion described earlier.

### Extracting terms from the corpus

We used RapidMiner, a framework for data mining analysis (https://rapidminer.com/) to obtain the terms frequently used in articles and that are important to consider in the biocuration. This tool contains different algorithms to generate a pipeline for extracting valuable information from a corpus. Our pipeline consisted of tokenizing all documents, transforming tokens to lower case, filtering the stop words and tokens of fixed length, and generating n-grams to make a union of consecutive words, with n from 1 to 5. We obtained 7,984 candidate terms from the corpus (see online supplementary Table S3). All the candidate terms were reviewed manually with the help of an expert committee (the coauthors of this article), and we focused mostly on those terms related to regulatory effects and characteristics of the samples. Terms related to regulatory effects were compared and expanded with those in the annotation dictionary used in our previous work with *Escherichia coli* (29), and terms related to characteristics of the samples were analyzed by the expert committee and incorporated in the dictionary.

### Annotation dictionary

The OntoGene/ODIN system, in its most basic usage mode, simply annotates specific entities in a target document, relying on a terminological dictionary provided by the user. The user can then manipulate the annotated documents using the ODIN interface, and in particular, it is possible to ‘filter’ the document according to the presence of specific entity types. For example, the user can choose to view only sentences containing annotated terms of a given type.

We created a list of terms to be used by the OntoGene/ODIN system as an annotation dictionary to facilitate the use of ODIN filters (see online supplementary Table S4). We added the names of miRNAs that have been found experimentally validated with strong evidence in miRTarBase (4), those with high confidence in miRBase, including all its naming conventions (19), and all the names of human genes that followed the HUGO nomenclature (34). It was important to add several databases since there are different nomenclatures for describing miRNAs (19, 34, 35) and many variations of the names appear in published articles. For instance, in the article with PMID 20643828 (36) ‘miR-21’ appears, while in HUGO it is written as ‘MIR21’, and other forms could be found as ‘hsa-mir-21’, or ‘hsa-mir-21-5p’. OntoGene is capable of recognizing some variants of the term, such as uppercase/lowercase changes, the introduction of punctuation signs (e.g. addition/removal of a hyphen between fragments of the term) automatically. It can also capture ‘pre-‘ and ‘pri-’ modifiers.

Additionally, we included different species, in case they could be mentioned in relation to human miRNAs, e.g. mouse and rat; and, the names of all the TFs present in the database for eukaryotes to differentiate structural and regulatory genes (37). Finally, terms related to effects and characteristics of the samples were taken from the terms previously obtained with the RapidMiner pipeline. All the terms were classified into categories or types, i.e. miRNAs, pre-miRNAs, pri-miRNAs, Gene, Transcription factor, Organism, Disease, Regulatory Effect and Characteristics of the Sample (see Table 1). The annotation dictionary was built as generic as possible to facilitate the use or expansion of our platform with different organisms and diseases. An important feature of OntoGene/ODIN, is that it has an option where the biocurator can easily add new terms when they are not incorporated in the dictionary.

**Table 1.** Categories of terms used in the annotation dictionary

Term type	Source	Example(s)	Total terms in the dictionary by category
**MicroRNA** name	miRTarBase (5), miRBase (20), HGNC (Hugo Gene Nomenclature Committee) (34)	hsa-mir-21-5p, hsa-mir-21	32 548
Target **Gene**	HGNC (34)	SMAD7	165 849
**Transcription factor**	Jaspar (37)	ZEB1	1191
**Organism**	NCBI taxonomy (41)	Human, rat, mouse (different species)	174
**Disease** associated	Terms reviewed by experts	IPF	26
Level of microRNA under some conditions, or the regulatory **Effects**	Previous work (31), and RapidMiner pipeline	Up, down, overexpressed, deleted, induced, repressed	90
Characteristics of the **Samples**	RapidMiner pipeline and terms reviewed by experts	Type of sample (lung tissue, alveolar macrophages, fibroblasts) Characteristics of the samples or **Variables:** Gender (female, male) Age Condition (normal *vs.* disease) Smoking status (current, former, past) Race (Caucasian, Afro-American, etc.)	124

### Using regular expressions for miRNAs

Since the names of miRNAs appear at first sight to have quite a regular structure, it would seem possible to capture all or most of them with regular expressions. As a matter of fact, we found that the names of miRNAs are more complex than it appears at first sight. However, the main disadvantage of using regular expressions is that we would still be left with the task of associating a database identifier to any recognized miRNA name, while our dictionary-based method automatically delivers also the identifier whenever a name is recognized. Besides, an approach based on regular expressions might deliver miRNA names that are not yet in any database. At that point, it might be difficult to decide if such a case corresponds to a new name or it is a false positive.

In any case, to further explore this application, we have written regular expressions that can capture all the currently existing miRNA names. We provide the complete set of regular expressions as online supplementary material S5. However, the core expression can be summarized as follows:

((pre|pri)-)?**prefix**?**mirna**-?(iab-)?[0-9]{1,4}**value**?**suf1**?**suf2**?

Where the parts in boldface correspond to:

**prefix**="(ath|bmo|bta|cel|cfa|dme|dre|ebv|gga|hcmv|hsa|hsv1|hsv2|mml|mmu|ola|ptr|rno|ssc|tgu|xla|xtr)-)"

**mirna**="(mir|miR|MiR|MIR|MIRN|MIRLET)"

**value**="[A-Za-z]?[0-9A-Za-z]?[0-9]?(HG)?"

**suf1**="(-[1-9][0-9]?|-[123]HG)"

**suf2**="(-3p|-5p)"

This expression has been designed to capture most of the miRNA names, yet avoid capturing any false positives. This is achieved by requiring the presence of one of the **mirna** strings, followed by at least one digit.

As a matter of fact, some of the miRNA names do not conform to this pattern. To capture all of them, we had to introduce a few additional more specialized regular expressions. The final set has been tested on all miRNA names obtained from HGNC, miRBase and miRTarBase (31 905 terms), and we have verified that they capture all of them. To verify that the expressions do not capture false positives, we have applied them to a corpus of 5 755 731 words obtained from the Leipzig Corpora Collection (http://corpora.uni-leipzig.de/en, corpus “eng-uk_web_2002_300K-sentences”). Since this corpus contains non-biomedical text, it is very unlikely that it might contain a miRNA name. In fact, the regular expressions did not provide any match. Based on this experiment, we could deduce that our regular expressions have 100% precision and 100% recall. However, it is, of course, possible that new miRNA names, which do not conform to our set of regular expressions, will be used in an article (reducing recall). It is also possible that words not contained in our test set are matched by our regular expressions, even if they are not miRNAs (reducing precision).

In conclusion, for our specific application, regular expressions do not provide currently any advantage over the dictionary approach, therefore we have not used them in the rest of the experiments described in this article, though we reserve to use them for future experiments.

### Biocuration process

Articles were processed by OntoGene with the annotation dictionary generated earlier and then visualized using the ODIN platform (http://odin.ccg.unam.mx/ODIN/bc2015-miRNA/). Biocuration was performed through assisted curation using OntoGene/ODIN and also by the traditional method—reading articles and annotating data related to miRNAs manually to make a comparison between these two approaches. Screencasts and detailed user manual documentation of OntoGene/ODIN are accessible to the biocurators from a link within the system. In the manual biocuration, we included other articles that were not freely available. We also reviewed the experiments in figures and online supplementary material. The list of curated miRNAs is provided in online supplementary Table S6.

## Results and discussion

### Filters design allows finding relevant sentences for curating microRNAs

**Figure 1.**
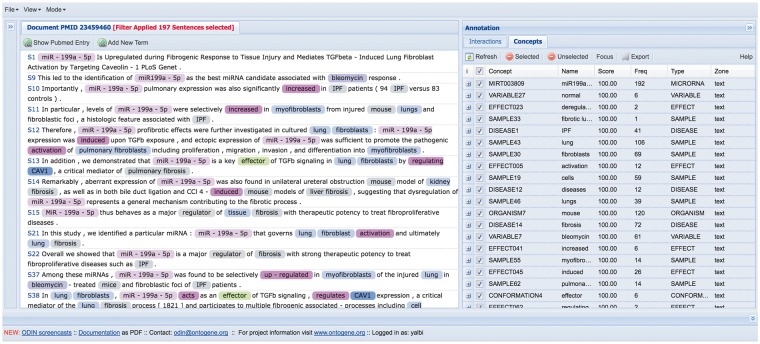
ODIN interface. At the left side there is the article tagged and at the right side all the terms that appeared at the dictionary.

ODIN provides an interface in which the biocurator can read full text, or only the relevant sentences of the article using some filters (see Figure 1, ODIN interface). By ‘relevant’, we mean sentences including user-selected terms from the dictionary that have been previously created. These terms are also highlighted, for easier navigation.

When the curators start their work, the ODIN interface presents the complete document (full text), so the curators can read all the article if they want. During their work, they can apply filters to diminish the number of sentences to read, after this, they can return to the full text if they have any doubt in understanding a sentence or reading an entire paragraph. They can choose among a small set of predefined filters, or define their own filters. (See Table 1: categories of terms used in the annotation dictionary).

For example, the most general procedure to find the information which we need to curate is to use only the concept ‘MICRORNA’ as a filter in the View menu. The MICRORNA concept selects all terms which have been annotated as miRNAs (see ‘Methods’ section). There was a total of 26 347 sentences in the original corpus, and with the filter, only 2799 remained, which represented 10.62% of the corpus.

In ODIN, it is possible to reduce the number of sentences to be read applying some rules to select more carefully the information. Sentences in scientific articles have a semantic structure that can be used to retrieve interactions or conditions (29). We applied Boolean operators (AND, OR) to find the relevant sentences and obtain the semantic structures. Rules can be more relaxed or stricter depending on the level of information that we want to curate, and this is reflected in the number of sentences, which are reduced when we apply more rules.

For our purpose, we were focusing on sentences, which expressed the following conceptual structures:

- MicroRNA (Effect)? (AND Disease)? Example: miR-185 is increased in IPF.
- MicroRNA (Effect)? AND TargetGene (AND Disease)?

  Example: An increase in miR-21 targets Smad7 in IPF.

- MicroRNA (Effect)? AND TargetGene (AND Sample)? (AND Disease)? Example: There is an increase in COL1A2 (A MIR-29b target) in lung biopsies from IPF patients.

For example, in the last case, *MIR-29 b* is the MicroRNA, ‘increase’ is the Effect, *COL1A2* is the TargetGene, ‘lung biopsies’ is the sample and *IPF* is the disease. A similar approach was previously used in (29) to extract transcription factors and other elements relevant to *Escherichia coli* regulation. In our case, the use of filters allows customizing our search depending on what we are looking for. If we want to find sentences to know if a determined miRNA is induced or repressed in IPF, we apply ‘MICRORNA’ AND ‘EFFECT’ = ‘induced or repressed’ AND ‘DISEASE’ = ‘IPF’ filters.

In the examples shown in online supplementary Table S7, some key terms indicated effects, i.e. positive or negative changes (increase, decrease, induction, repression) in levels of expression of the microRNA or levels of its target gene. When a sentence contains these terms, it is very common to observe the experimental conditions (disease *vs.* control) or cell types (fibroblasts, epithelial cells etc.)

**Figure 2.**
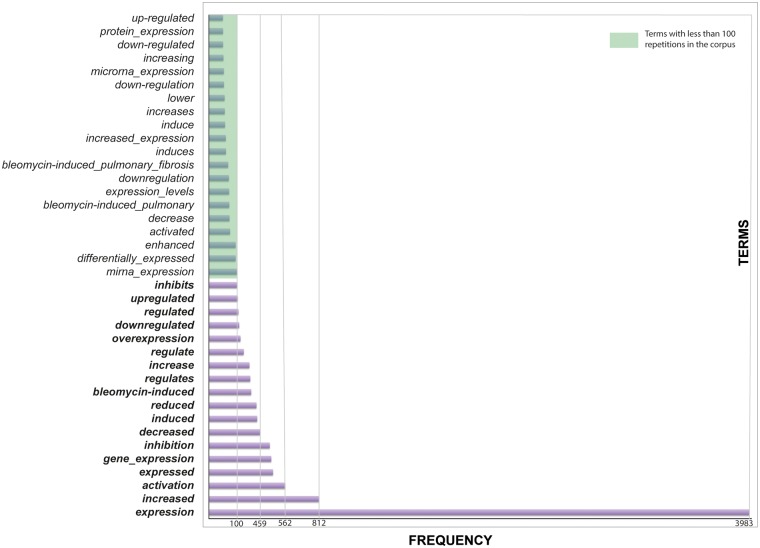
Terms related to effects. Effects are important to indicate the level of the miRNA or the relation with the target gene.

Interestingly, in our corpus, terms that appeared more frequently are those that mean an induction or activation (Figure 2). This bias could be explained by the fact that there were more sentences related to the level of expression of the miRNAs than about the interaction of the miRNAs with their target genes.

Some words are related to no changes in effects, e.g. ‘miR-338 (miR-338-5p) was found to be significantly increased (Figure 6A) while miR-99 b and miR-323 ‘remained constant’ (data not shown)’ (38). Although this sentence is also giving us valuable information, it is not so common to find sentences where there are no changes in the level of gene/microRNA expression. Certainly, when there is no change in expression, it is not normally reported.

**Figure 3.**
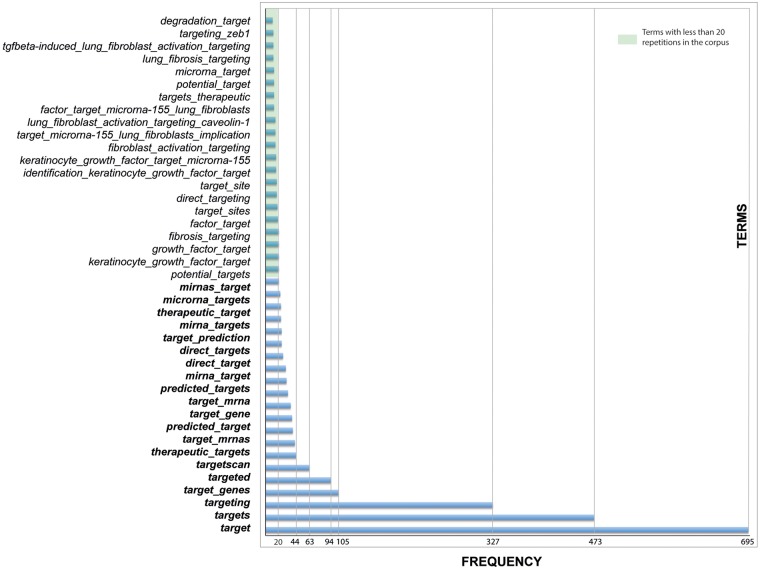
Terms used to find miRNAs and their target genes.

Besides, we found some key terms to be more accurate in obtaining target genes of miRNA—of course, the most important was the word ‘target’, as noun and verb (to target, with all its verbal forms)—they appeared very frequently in the corpus, alone or combined with other terms (Figure 3). In general, it can be expected that when the term ‘target’ appears in a sentence, it contains the interaction between the microRNA and its target gene (10) (see an example in online supplementary Table S7).

Our use of main terms annotated in the dictionary, allowed us to create filters and obtain levels of miRNAs in specific conditions and the interactions with their target genes (see online supplementary Table S6).

### The importance of incorporating sample information in biocuration

Although there are different tools to extract miRNAs from the literature, we selected OntoGene/ODIN because of previous successful results in finding the regulatory elements in the database RegulonDB (29), the usability of the system, the possibility of easily personalizing a different corpus of articles and only adapting the annotation dictionary, and the availability of the sentence filters which enable more efficient biocuration.

One of the reasons for which we wanted to personalize OntoGene is the necessity of incorporating information about the conditions under which miRNAs participate in IPF. It is well known that expression of miRNAs can be different depending on the cell type, organism and other characteristics of the samples. It is important to curate not only interactions but also the conditions and cell types where miRNAs have an effect.

For example, the following sentence mentions the samples or cell types where experiments were done:

‘miR-200a, -b, and -c are **downregulated** in **mice** with **lung fibrosis**; miR-200a and -c are **reduced** in **lungs** of **patients** with **IPF**; miR-200 had **higher expression** in **alveolar epithelial cells** (AECs) than in **lung fibroblasts,** and AECs from **mice** with **pulmonary fibrosis** had **diminished** expression of miR-200’ (39).

However, characteristics of the samples are difficult to obtain for several reasons, e.g. they are frequently shown in figures, tables or online supplementary material and most of them have to be curated manually; they are mentioned a few times in a article, and not all the variables are referred to in the article. With our protocol, we realized that using the filter *OR* allowing us to include more sentences in the same search is useful to find sample information, e.g. in online supplementary Table S5, the age, and gender of the patients are mentioned, factors that are important in IPF (17). Combining manual biocuration with our semiautomatic method, allows us to gain quality, paying attention to false positives/negatives, covering most of the information that needed to be curated.

As a result, using this semiautomatic pipeline, we could curate 823 miRNA text snippets for a total of 246 miRNAs associated with IPF.

### BioCreative V IAT track insights

There have been several ‘challenges’ in which the text mining community compared their methods to see the scientific progress in that field. The BioCreative IAT was designed specifically for exploring user-system interactions, between the biocurators and the text mining communities (31). Typically, the IAT does not have a quantitative evaluation like the other BioCreative tasks but investigates the usability of biocuration systems and uses a qualitative assessment based on detailed surveys answered by the biocurators.

The results of the IAT track are presented in detail in (25). The track centered on the usability evaluation of web-based systems addressing different curation tasks, performed by expert biocurators. Seven different systems participated in the track, each addressing different aspects of a biocuration pipeline for more details, see (31).

The OntoGene/ODIN system had a very high rate of completion and received the top scores on the SUS, usability and learnability. Also, the biocuration throughput improvement was the highest reported: 12 articles per day, as opposed to 1 article per day without the assistance of the system. This comparison is based on curation efforts performed for the experiments described in this article, which however used less experienced curators, who could not manage to curate more than one article per day using the traditional approach (without automated support). However, even in the case of the experienced curators of the RegulonDB database, no more than two articles per day can be achieved with the traditional approach (29). In conclusion, even in the case of experienced curators, the biocuration throughput improvements can be expected to be at least a factor of six.

## Concluding remarks

When compared with the standard approach based on manual biocuration, the semiautomated approach described in this article allows us to save a considerable amount of time. The approach does not replace the activity of the biocurator but makes it more efficient: to truly understand and annotate the interactions, the expertise of the biocurator remains essential. However, semiautomated biocuration has the key advantage that it significantly reduces the number of sentences to be read by the curators, thus providing a major competitive advantage. A biocurator reads about one article per day; this means that the 66 articles with manual biocuration are completed in ∼2 months. Using ODIN, the time required for this work was reduced to only 5 days. This notable increase in efficiency is the major methodological contribution of this work. It is well known that a major bottleneck in accelerating the processing of the huge amount of knowledge generated in biomedical sciences is the curation and encoding of knowledge presented in publications into structured and computer-accessible databases. One disadvantage of our pipeline is that information contained within figures, tables and online supplementary materials are not yet extracted by the system. Adding them will be an interesting challenge for future work. An important advantage is that our system can be extrapolated easily to other diseases, with a simple adaptation of the annotation dictionary.

Finally, our semiautomatic approach retrieved 246 miRNAs associated with IPF that corresponds to 100% of IPF-associated miRNAs that can be obtained manually in this corpus, so we expect similar good results when we apply this protocol to other respiratory diseases. However, notice that this is the precision of the entire process: automated annotation followed by manual curation. Thus, such a high precision is not surprising, since our semi-automatic process includes the participation of curators, which are those who have curated our gold standard information against which we evaluate precision.

## Supplementary data

Supplementary data are available at *Database* Online.

## Supplementary Material

Supplementary Data
